# Preventive healthcare uptake in private hospitals in Nigeria: a cross-sectional survey (Nisa premier hospital)

**DOI:** 10.1186/s12913-020-05117-5

**Published:** 2020-04-01

**Authors:** Joshua N. T. Ofoli, Timi Ashau-Oladipo, Stephen S. Hati, Lile Ati, Victor Ede

**Affiliations:** 1 Department of Family and Specialty Medicine, Nisa Premier Hospital, Abuja, Nigeria; 2 Research and Development, Nisa Premier Hospital, Abuja, Nigeria; 3 Center for Research, Institute of Medical Sciences Africa, Abuja, Nigeria; 4 Medical Audit Department, Nisa Premier Hospital, Abuja, Nigeria

**Keywords:** Preventive healthcare, Characterization, Nisa hospital, Abuja, Private hospital, Primary healthcare, Health insurance cover

## Abstract

**Background:**

Understanding the features of preventive care uptake is critical for assessing the performance and viability of primary care in any healthcare system. There are gaps in previous studies that focused on primary healthcare features, challenges and way forward in Nigeria but were mainly public sector focused and do not characterize the features of preventive care. Since private healthcare sector remains the most accessed and utilized in Nigeria, this study sought to characterize the features of uptake of preventive care to better understand the current preventive healthcare landscape.

**Method:**

A descriptive cross-sectional study, using survey questionnaire were randomly administered to adult patients attending the Family Medicine Out-Patient Department (OPD) at Nisa Premier Hospital, Jabi Abuja. The study was conducted over a three-month period. (January to June 2017). Data collected were analyzed using SPSS version 23 (IBM SPSS, Chicago, IL, USA). Descriptive statistics in the form of frequency and percentage were used to report the results.

**Results:**

A total of 381 participants completed the survey. The results revealed that while an over overwhelming majority (> 90%) of participants indicated knowledge of benefits of preventive care, and preferred interventions aimed at preventing a disease before they occur, 48% preferred interventions aimed at reducing disease or injury impact or interventions aimed at ameliorating the impact of ongoing disease or injury with long lasting effect (43%). Unfortunately, less than 40% of respondents would visit the hospital when their health condition is not serious. Important barriers to uptake of preventive care were revealed as cost (45%), distance to the healthcare provider (36%) and lack of health insurance (33%), whereas poor education (19%), social norms (13%) as well as cultural and religious beliefs (10%) towards accessing certain health services appeared to be lesser barriers.

**Conclusion:**

Although people are aware of the benefits of preventive care, its uptake will greatly be enhanced through improved health insurance coverage, refocusing primary healthcare functions on preventive rather than curative care and instituting policies that mandatorily prescribe uptake for the insured, both at the individual and the insurer’s level.

## Background

Disease and disability are dynamic processes which begin before individuals realize they are affected; therefore prevention of diseases largely relies on anticipatory actions which are termed preventive care [1]. It consists of measures taken for disease prevention, as opposed to disease treatment [1]. Preventive care services encompass a wide range of healthcare measures including routine check-ups, disease screenings, and immunizations, which can be undertaken to prevent the occurrence of disease and detect disease early [2, 3].

Prior studies have shown that preventive care service utilization reduces premature mortality and improves quality of life [2, 4]. Underutilization of preventive care services may result in failures to identify treatable healthcare problems and prevent potentially life-threatening diseases [4]. According to estimates made by the World Health Organization (WHO), about 55 million people died worldwide in 2011, with two thirds of this group dying from non-communicable diseases and 60% of these deaths were attributed to preventable disease. Preventive healthcare is especially important, given the worldwide increase in the burden of chronic diseases and preventable deaths.

However, despite the clamor and advocacy for prevention, uptake of preventive health services is still poor [5, 6] owing to different factors such as age, income, insurance coverage, awareness, access to preventive services [7]. Many countries have adapted preventive care models, or are promoting preventive health services on the premise that prevention does not only improve health, but also has a cost-saving potential or confers substantial health benefits relative to their cost [8]. Researchers have argued about the effectiveness of applying a preventive measure towards the general population rather than direct prevention towards a target population [9]. Others refute this claim, stating that a particular preventive measure termed to give good value or poor value depends on factors such as the population targeted, with measures targeting higher-risk populations typically being the most efficient [8].

In addition, studies have shown that the factors that determine utilization apply differently to different settings; this was proven by exploring the inequality in utilization of preventive care services using different variables. For example, Chen et al. [9] and Ayanda [10] noted that rural residents were less likely to utilize preventive care services when compared to urban residents in China and Nigeria respectively. Conversely, Howe et al. identified existing age, race, income, and insurance status-related disparities in preventive care utilization within a US population [11]. One study also explored the private and public health sectors in health care delivery by comparing the services rendered in both sectors including preventive care [12]. The study reported that in Colombo, Sri Lanka, the private sector provided more than 72% of all childhood immunizations, among a population. Similarly, the Nigerian health system is a blend of both private and public health care with relatively more people seeking care in private settings for most of their health demands due to various reasons such as travel time, education, age, sex, level of education of household head, household size and perceived quality of care provided in the facility [13]. Considering these various factors, it will be important to explore utilization of the preventive health services provided in private hospital settings as this determines the success of any health delivery system. Therefore, in this study we sought to characterize the uptake of preventive healthcare and determine the rate of preventive care utilization in private hospitals in Nigeria. A number of studies have considered primary healthcare challenges and way forward in Nigeria [14, 15], but are mainly public sector focused and do not characterize the preventive health care landscape which is critical to primary healthcare. Since private health sector is the most accessed and utilized in Nigeria, characterizing the uptake of preventive health care will provide understanding of the available infrastructure, utilization, and barriers to preventive healthcare access.

## Methods

### Study design

This cross-sectional survey research was conducted from June 2017 to February 2018. Paper questionnaires were administered by medical receptionists, patient care assistants and quality assurance officers in the family medicine Out-Patient Department (OPD). They were trained on the process of questionnaire administration for a period of 1 week. A representative convenience sample of patients, doctors, nurses, and support staff from the OPD were recruited into the study. The OPD offers emergency care services, outpatient visits, immunization, preventive care services, laboratory, radiological and drug refill services. The preventive healthcare services available in the hospital include pre-employment, pre-school, women-centered, child wellness, and diabetic packages.

### Survey frame

According to Wada et al. (2016), the case study, Nisa Premier Hospital, has in its database over 50,000 registered patients as at 2014 [15]. In adopting the sample frame, the monthly average number of visits to the Family Medicine OPD which constitutes 70% of visits per month in a polyclinic setting was utilized.

The sample size was calculated with a confident level of 95% and a confidence interval of 5% a sample size of 346 respondents from a total sample frame of 3500. The confidence interval of 5% suggests that between 45 and 55% of the study population would provide the answers that were obtained from this study. The attrition rate was set at 10% of sample size (35), which gave a total sample size of 381. Questionnaires were administered to a total of 586 adults. There was a 65% response rate; hence 381 completed questionnaires were used for analysis. Informed consent forms were reviewed and approved by the Nisa Scientific Review Committee. Written informed consent to participate in the study was obtained from all participants prior to participation.

### Inclusion criteria

The inclusion criteria included participants between ages 18 years to 65 years, visiting the OPD as patients or staff of the hospital. Both males and females who were literate and medically stable were invited to participate.

### Exclusion criteria

Children below the age of 18 and older adults above 65 years, in-patients and the very ill patients of other sub specialties were excluded along with patients who could not give consent.

### Survey instrument

Due to a paucity of questionnaires on this subject in the region of interest, the researchers developed their own questionnaire using relevant books, studies and references and in accordance with the Theory of Planned Behavior (TPB). The questionnaire included multiple choice questions relating to factors that affect preventive health care uptake (see Supplementary file 1). The questionnaire asked the participants’ demographic information, including age group, gender, level of education, marital status, employment, monthly income, distance from nearest health clinic/hospital, and examined the constructs of TPB; including attitude, subjective norms, perceived behavioral control, behavioral intention and behavior. Cronbach’s alpha values were calculated to confirm the reliability of each of the constructs.

Behavioral intention (nine items), attitude (two items), subjective norms (two items) and perceived behavioral control (five items) were assessed based on a five-point Likert scale with responses ranging from 1 (never) to 5 (every 5 years); four-point scale ranging from 1 (life threating) to 4(not serious); and ‘yes’ (1 point) and ‘no’ (0 points) responses. Ultimately, behavior was assessed with 22 items, one of which was scored based on the Likert scale and the remaining 21 by ‘yes’ (1 point) and ‘no’ (0 points) responses.

The reliability of the items pertaining to the constructs of the TPB was confirmed by measuring their Cronbach’s alpha, which was reported as α = 0.74 for attitude, α = 0.74 for subjective norms, α = 0.74 for perceived behavioral control, α = 0.74 for behavior and α = 0.74 overall. The questionnaire took 15 min to be completed.

Questions asked to determine health seeking behavior of respondents include “Where do you usually go if you are sick or to treat a general health problem?” which had the following options private hospitals, general hospitals, drug store and natural healers, “How often do you seek health care at a clinic or Hospital?”, whether monthly, half yearly, annually or every 2 years, and a question on the severity of an illness before seeking care where the options included life threatening, very serious, somewhat serious, and not serious.

We assessed adoption of preventive health measures using the following questions: (1) Are you aware of the term preventive health care? (2) Which aspect of preventive healthcare do you prefer? Options varied from: Interventions aimed at preventing a disease before they occur; Interventions aimed at reducing disease or injury impact; Interventions aimed at ameliorating the impact of on-going disease or injury with long lasting effect; All and none of the above. (3) Which of the following constitute preventive health care? The option include Preventive health check-ups/physical exams, Regular age related screening tests, Lifestyle- related advice like diet/nutritional counseling, Facilities for exercising like gym or park, Stress-relieving techniques like Yoga, and Routine vaccinations (4) How often or at what interval do you undergo preventive health check-up? (5) What preventive health measure do you take? Amongst Immunization, Insecticide Treated Nets, Exercise, Dieting, Stress management, Weight control, Yoga and Prophylaxis? (6) Which preventive healthcare services have you accessed in the past 2 years? (7) Which of these would prevent you from accessing preventive health care? With options including Poor access/distance to the healthcare providers, Cultural and religious beliefs, Underutilization of available health information, Inadequate/lack of Health insurance coverage, Stigma and social norms towards accessing certain health services, Inadequate education on benefits of Preventive Healthcare and Cost of healthcare (see Supplementary file 1). Questions 1 and 3 assessed knowledge of preventive healthcare while questions 4, 6 and 7 were used to assess the uptake of preventive healthcare services. Responses to knowledge and uptake of preventive healthcare constructs were categorized and coded into yes or no. The survey instrument was piloted among 20 staff at a large partner hospital in Abuja for a period of 1 week. The staff’s feedbacks were compared for consistency and redundancy. Additional domains of interest were also identified. The survey was further evaluated for relevancy, clarity and appropriateness of responses by four more staff. Modifications were made to the survey in response to this review.

Paper data were collected and entered on an excel sheet. Analysis was done using SPSS statistical package version 23 (IBM SPSS, Chicago, IL, USA). Descriptive statistics in the form of frequency and percentage were used to report the results. Differences in responses by participant demographic characteristics were determined using the 2 – sided Chi-square test.

## Results

A total of 381 participants completed the survey. The participant’s ages ranged from 18 to 65 years with the majority (80.0%) between the ages of 25 and 44 years (Table 1). In terms of gender, 61.0% of the subjects were female. The majority (98%) of the participants had attained either tertiary or postgraduate education. About 78.0% of the participants were married mothers and more than half (55%) of the participants estimated their monthly income at 100,001–400,000 naira. As shown in Table 1, over 70% of the participants lived within 10 km from a clinic or hospital. Seventy-seven percent of participants indicated that they usually seek health care in a private hospital. Table 2 states that only 13% of participants go to public hospital when they are sick. Regarding factors that determine choice of hospital, the majority of participants (74%) chose a hospital based on the availability of specialized doctors and qualified health staff. Almost half of the participants indicated that the ease of accessing care, wait time to receive care (45%), staff courtesy/friendliness (42%) and the cleanliness of the hospital (43%) all influence their choice of hospital (See Table 2).

**Table 1:** Demographic characteristics

Characteristics	N (%)
**Age (Years)**	
18–24	29 (8)
25–34	176 (46)
35–44	131 (34)
45–54	36 (9)
55–65	9 (2)
**Gender**	
Female	231 (61)
Male	150 (39)
**Highest level of education**	
Postgraduate	214 (56)
Primary	1 (0)
Secondary	5 (1)
Tertiary	161 (42)
**Marital Status**	
Married	297 (78)
Separated	3 (1)
Single	81 (21)
**Type of Employment**	
Unemployed	11 (3)
Government	114 (30)
Non-Governmental Organization	1 (0)
Private	183 (48)
Self-employed	72 (19)
**Monthly Income (₦)**	
< 100,000	117 (31)
100,001–400,000	209 (55)
400,001–1,000,000	39 (10)
> 1,000,000	16 (4)
**Distance from nearest hospital**	
> 15 km	50 (13)
≤ 5 km	166 (44)
11-15 km	39 (10)
6 - 10 km	126 (33)

Note: *km* Kilometer; *₦* Naira

**Table 2:** Health seeking behavior

Variables	N (%)
**Place of choice to go to for illness/treatment**	
Drug store (Pharmacy)	37 (10)
Government hospital	50 (13)
Private Clinic	293 (77)
Others	1 (0)
Traditional healer	0 (0)
**Frequency of hospital visits**	
Every 6 months	147 (39)
Every 1 year	68 (18)
Monthly	113 (30)
Two years above	37 (10)
When there is need	16 (4)
**Factors that determine choice of hospital**	
0Proximity	111 (29)
Working hours of health facility	67 (18)
Staff courtesy	159 (42)
Qualified health staff	281 (74)
Waiting time	171 (45)
Cleanliness of facility	162 (43)
Reputation of health facility	144 (38)
Cost of services	83 (22)
**Extent of illness before visiting healthcare facility**	
Life threatening	10 (3)
Not serious	150 (39)
Somewhat serious	193 (51)
Very serious	28 (7)
**Diseases that have required hospital intervention in the past 1 year**	
Acute diseases	263 (69)
Infectious diseases	18 (5)
Chronic diseases	49 (13)
None of the above	32 (18)
Others	34 (9)

In assessing the seriousness of an ailment prompting visit to a health facility, about half (51%) of the participants would wait until their condition was somewhat serious. However, 39% of participants mentioned that they would visit the hospital when their health condition is not serious (Table 2). With regard to awareness of the term preventive health care, 87% of the participants knew what preventive health was. Table 3 depicts that the overwhelming majority (91%) of participants preferred interventions aimed at preventing a disease before they occur whereas near half preferred interventions aimed at reducing disease or injury impact (48%) or interventions aimed at ameliorating the impact of ongoing disease or injury with long lasting effect (43%). When asked what preventive health measures they take, 61% mentioned exercise, followed by immunization (48%), and dieting (41%). Over the past 2 years, blood pressure (65%), blood sugar (54%) and weight (49%) checks were the top 3 preventive health care services accessed by participants. Access or utilization of age-specific and gender-specific preventive healthcare services such as pap smear (15%), colon cancer screen (1%), prostate cancer screen (4%), chlamydia screening (3%) and mammography (5%) were low likely due to our participant demographic (see Table 4).

**Table 3:** Awareness of preventive health care

Variables	N (%)
**Awareness**	
No	51 (13)
Yes	330 (87)
**Source of awareness**	
Mass media	113 (30)
Social media	114 (30)
Health workers	141 (37)
Family friends	99 (26)
Religious/Community leaders	20 (5)
Others	44 (12)
**Preferred aspect of Preventive healthcare**	
Intervention aimed at preventing a disease before they occur	348 (91)
Interventions aimed at reducing disease or injury impact	182 (48)
Interventions aimed at ameliorating the impact of ongoing disease or injury with long lasting effect	163 (43)
**Preventive health care components**	
Preventive health check-ups/physical exams	318 (83)
Regular age related screening tests	259 (68)
Lifestyle-related advice like diet/nutritional counseling	277 (73)
Facilities for exercising like gym or park	239 (63)
Stress-relieving techniques like Yoga	201 (53)
Routine vaccinations	260 (68)
Others	4 (1)
**Preventive health checkup interval**	
6 monthly	138 (36)
Annually	122 (32)
Every 5 years	4 (1)
Every 2 years	20 (5)
Never	97 (25)
**Participant preventive health care measures**	
Immunization	181 (48)
Insecticide treated net	114 (30)
Exercise	232 (61)
Dieting	155 (41)
Stress management	116 (30)
Weight control	131 (34)
Yoga	16 (4)
Prophylaxis	27 (7)

**Table 4:** Uptake of preventive health care services

Variables	N (%)
**Preventive health care services accessed in past 2 years**	
Pap smear	57 (15)
BP check	249 (65)
Blood sugar	207 (54)
Cholesterol/lipids	90 (24)
Screening for STIs (Including HIV)	162 (43)
Screening for Hepatitis B & C	147 (39)
Chlamydia screen	10 (3)
Colon cancer screen	4 (1)
Prostate cancer screen	17 (4)
Dental screen	85 (22)
Eye care	124 (33)
Weight check	188 (49)
Others (specify)	5 (1)
**Barriers to preventive health care access**	
Poor access/distance to the health care providers	137 (36)
Cultural and religious beliefs	40 (10)
Underutilization of available health information	66 (17)
Inadequate/lack of health insurance coverage	127 (33)
Stigma and social norms towards accessing certain health services	49 (13)
Inadequate education on benefits of preventive healthcare	74 (19)
Cost of health care	173 (45)
None of the above	34 (9)
Others (please specify)	4 (1)
**Preferred preventive health care packages**	
Domestic staff screening	99 (26)
Pre-employment test	91 (24)
Preschool screening test	72 (19)
Advanced heart check	78 (20)
Diabetic package	73 (19)
Liver screening package	75 (20)
Pre-marital screening	98 (26)
Routine complete check-up	254 (67)
Senior citizens check-up	36 (9)

As for barriers to access to preventive healthcare shown in Tables 4, 45% of the participants indicated that the cost of healthcare prevented them from accessing preventive health care. Among study participants, high healthcare cost was the most frequent barrier to preventive care access followed by distance to the healthcare provider (36%) and lack of health insurance (33%). Whereas cultural and religious beliefs (10%), stigma and social norms towards accessing certain health services (13%), and inadequate education on the benefits of preventive healthcare were lesser barriers to accessing preventive health care (19%). Of the preventive healthcare packages offered, the majority (67%) of participants indicated that they would access the ‘routine complete checkup package’.

We observed significant differences in demographics relative to knowledge and uptake of preventive healthcare. Table 5 show that females (57.8%) were more likely to have knowledge of the components of preventive healthcare compared to males (*p* < 0.001). Individuals who were married frequently accessed preventive health care than those who were single or unmarried. The age ranges 25–34 years and 35–44 years were more likely to frequently access preventive health care and had in the past 2 years accessed a preventive healthcare service than were other age groups. Compared with other forms of employment, privately employed individuals (50.6%) were more likely to have knowledge about the preventive health care services available to them.

**Table 5:** Cross-tabulation of demographic characteristics and preventive healthcare services uptake

	Knowledge of Preventive Healthcare				Utilization of Preventive Healthcare Services					
	Awareness of Preventive Healthcare		Knowledge of the components of Preventive Healthcare		Frequency of Preventive Healthcare Visits		Two-Year Uptake of Preventive Healthcare Services		Future Planned Access to Preventive Healthcare Services	
	N (%)	*p*	N (%)	*p*	N (%)	*p*	N (%)	*p*	N (%)	*p*
**Age**		0.217		0.012		0.012		0.014		0.259
18–24	22 (6.7)		23 (6.6)		16 (5.6)		21 (6.2)		24 (7.1)	
25–34	159 (48.2)		154 (44.5)		129 (45.4)		155 (45.6)		152 (44.8)	
35–44	111 (33.6)		125 (36.1)		98 (34.5)		121 (35.6)		121 (35.7)	
45–54	30 (9.1)		35 (10.1)		32 (11.3)		35 (10.3)		33 (9.7)	
55–65	8 (2.4)		9 (2.6)		9 (3.2)		8 (2.4)		9 (2.7)	
**Gender**		0.981		0.000		0.06		0.771		0.858
Female	200 (60.6)		200 (57.8)		180 (63.4)		207 (60.9)		205 (60.5)	
Male	130 (39.4)		146 (42.2)		104 (36.6)		133 (39.1)		134 (39.5)	
**Education Level**		0.079		0.091		0.108		0.403		0.134
Postgraduate	187 (56.7)		193 (55.8)		167 (58.8)		195 (57.4)		188 (55.5)	
Primary	0 (0)		1 (0.3)		1 (0.4)		1 (0.3)		1 (0.3)	
Secondary	4 (1.2)		3 (0.9)		5 (1.8)		5 (1.5)		3 (0.9)	
Tertiary	139 (42.1)		149 (43.1)		111 (39.1)		139 (40.9)		147 (43.4)	
**Marital Status**		0.615		0.476		0.018		0.739		0.413
Married	255 (77.3)		272 (78.6)		230 (81)		266 (78.2)		266 (78.5)	
Separated	3 (0.9)		3 (0.9)		3 (1.1)		3 (0.9)		2 (0.6)	
Single	72 (21.8)		71 (20.5)		51 (18)		71 (20.9)		71 (20.9)	
**Employment Status**		0.009		0.112		0.443		0.108		0.242
Applicant	7 (2.1)		8 (2.3)		8 (2.8)		8 (2.4)		9 (2.7)	
Government	99 (30)		105 (30.3)		88 (31)		100 (29.4)		104 (30.7)	
Private	168 (50.9)		172 (50.4)		130 (45.8)		171 (50.3)		167 (50.2)	
Self-employed	56 (17)		62 (17.9)		58 (20.4)		61 (17.9)		59 (17.4)	

## Discussion

In this study nearly half of the participants were employed by private entities and had high literacy levels which may not be reflective of the employment type and literacy rates of the broader population. However, the distribution of age, gender, and marital status variable is comparable to that seen in the general population. We also found that the majority of participants lived less than 10 km from the nearest health care facility where they seek care and these points of care were mostly private clinics.

Monthly income was mostly between 100,000 to 400,000 naira (279 to 1116 USD). Based on world bank poverty and equity data portal for Nigeria, all participants were above the upper middle-income class poverty line [16]. Thus, in contrast with the general population, the average income level of our study participants was not representative of the income of most (53.5%) Nigerians who earn 20,520 naira (57 USD) monthly. Our study did not assess the statistical correlation between participant’s income level and the uptake of preventive health service.

From this study, as with public health studies of most Sub-Saharan countries [17], there is a lack of universal health coverage with a limited range of services and benefits covered. Furthermore, as a growing number of low and middle-income countries commit to achieving universal health coverage, one key challenge is how to extend coverage to the private sector [18]. The majority of Sub-Saharan African population access health care in private healthcare facilities [19]. Similarly, our study revealed that the majority of participants sought primary care in private healthcare settings and paid out-of-pocket for their health care. This is because less than 10% of the total Nigerian population is covered by the National Health Insurance Scheme (NHIS) [20, 21]. The NHIS is the government agency charged with securing universal coverage and access to adequate and affordable healthcare in order to improve the health status of Nigerians [20]. For the covered 10%, incorporating preventive health care packages to the NHIS plans may go a long way in improving the uptake of preventive health care. This is on the basis of the National Health Act (NHA) that mandates a basic minimum health care package and provides funding for this package [22]. Even more imperative is the need to expand the NHIS coverage to include all eligible Nigerians who are currently not covered.

An important finding of our study is that the cost of health care is a significant barrier to the access of preventive health care. This is despite the relatively higher income levels of the study population when compared with the general population. We can therefore project that the impact of the high cost of healthcare may be higher in the general population. Addressing this cost barrier by ensuring that a third-party payer such as NHIS covers the cost of preventive health care may improve access. Eliminating the cost barrier will in the long term be cost effective for the Nigerian health system as it would have greater population benefit. Challenges stemming from NHIS-delayed reimbursements, cost recovery and low revenue for primary health care services are faced by private health providers [19]. Hence for-profit private health sector providers may be averse to offering comprehensive preventive care programs due to these problems. Having a population-wide intervention through the NHIS that expands coverage and offers incentives to the primary care providers to offer preventive healthcare as well as offset the cost to the facility would possibly improve motivation to participate in the scheme and make care more accessible [19]. In addition, since most participants in the study were employed in private settings, more should be demanded from the self-employed and other private employers to provide health care coverage or insurance for their employees through private healthcare funders. As with the Affordable Care Act (ACA), adopting incentivized and/or subsidized preventive screening and health promotion programs – the structure of which increase the likelihood that beneficiaries will make positive lifestyle and healthy behavior choices, may help improve access to preventive health care [23, 24]. The incentives may take the form of a lower premium for participation in preventive screening [25]. This may also include additional reimbursements to physicians to encourage implementation of preventive care guidelines.

Individuals who were privately employed appeared to be more aware of available preventive health care services. Furthermore, while majority of patients showed good knowledge of the term ‘preventive health care’, at least half of the patients yet mentioned that an illness should be somewhat serious before visiting a health facility. Focusing on interventions that enhances the translation of knowledge of preventive health to implementation behaviors might be important to improve uptake of preventive health. An innovative approach in our primary care facility would be develop an integrative model of care that offers preventive health care services to all patients who come to the OPD seeking care for other medical conditions. This is based on our findings that suggest that apparently healthy individuals normally seek care when somewhat or acutely sick. Age specific and risk factor modified preventive healthcare screen reminders may also improve physician implementation of integrated preventive care protocols.

## Conclusion

Our results suggest that there may be relative lack of knowledge and poor preventive healthcare uptake rates among middle-aged and above individuals in Abuja who need to access preventive health care. Improving population education on the benefits of preventive healthcare and access to especially age-specific preventive health care services may help overall uptake of preventive healthcare. This study also revealed that despite awareness by some individuals, they still would not access preventive health care services until they fall sick. We have tried to explore factors that influence their uptake of these services and have recommended ways to improve this uptake. However, the survey constructs of our study did not allow us to correlate the statistical significance of these factors as well as how and why they impact on the uptake of preventive healthcare. Our next step would focus on identifying these associations and correlations. Furthermore, a limitation of our study was the lack of extension to other private healthcare facilities which may have been more revealing. However, this study is the first to our knowledge to characterize preventive health care uptake in private hospital settings in Nigeria and further research that includes a broader patient base is needed to validate these findings.

## Supplementary information

**Additional file 1.**

## Data Availability

The datasets used and/or analyzed during the current study are available from the corresponding author on reasonable request.

## Abbreviations

OPD: Out-patient department
WHO: World health organization
TPB: Theory of planned behavior
NHIS: National health insurance scheme
NHA: National Health Act
ACA: Affordable care act
