# Supplementary material for: Preventive healthcare uptake in private hospitals in Nigeria: a cross-sectional survey (Nisa premier hospital)
Source: BMC Health Serv Res. 2020 Apr 1;20:273. doi: 10.1186/s12913-020-05117-5 (PMC7114808; doi:10.1186/s12913-020-05117-5)
Supplement: Supplementary file 1 — Additional file 1. [file 12913_2020_5117_MOESM1_ESM.docx]

**FACTORS AFFECTING THE UPTAKE OF PREVENTIVE HEALTHCARE SERVICES IN PRIVATE HOSPITALS: A CASE STUDY OF NISA PREMIER HOSPITAL, ABUJA**



**SURVEY QUESTIONNAIRE**



The Quality Improvement team at Nisa Hospital invites you to participate in this research study. We are interested in knowing the factors that influence the uptake of preventive healthcare services in private hospitals (Nisa Hospital as a case study). Your response will provide useful information for health policy makers to exact evidence based decisions for delivery of preventative health care services.

Involvement in the study is voluntary; please feel free to ask questions about the research.

All information will be kept anonymous and confidential.

Preventive healthcare refers to those interventions that are targeted at prevention, as opposed to disease treatment or curative interventions. It is broadly grouped into primary, secondary and tertiary interventions. Primary intervention are aimed at preventing diseases before they occur, secondary intervention deal with reduction of disease or injury impact, while tertiary intervention aims to ameliorate the impact of an on-going illness or injury with long lasting effect.



**Consent:** Ticking **“Yes”**, gives us your consent to participate in the survey: **Yes** [ ] **No** [ ]

**SECTION -A: BACKGROUND OF RESPONDENTS**

Please Tick [√] appropriately

**1. Age group (years)**

[ ] 18-24

[ ] 25-34

[ ] 35-44

[ ] 45-54

[ ] 55-65

**2. Gender**

[ ] Male

[ ] Female

**3. Highest level of Education**

[ ] No School

[ ] Primary

[ ] Secondary

[ ] Tertiary

[ ] Postgraduate

**4. Marital status**

[ ] Single

[ ] Married

[ ] Separated

[ ] Divorced

**5. Type of employment**

[ ] Self-employed

[ ] Private

[ ] Government

[ ] Applicant

**6. What is your monthly income level?**

[ ] <100, 000

[ ] 100,001-400,000

[ ] 400,001-1million

[ ] > 1million

**7. Distance from the nearest health clinic/hospital**

[ ] ≤5km

[ ] 6-10km

[ ] 11-15km

[ ] >15km

**SECTION -B: HEALTH SEEKING BEHAVIOUR**

Please Tick [√] appropriately

**8. Where do you usually go if you are sick or to treat a general health problem**

[ ] Private clinic

[ ] Government hospital

[ ] Traditional healer

[ ] Drug store (Pharmacy)

[ ] Others___________________

**9. How often do you seek health care at clinic or Hospital?**

[ ] Monthly

[ ] Every 6 months

[ ] Every one year

[ ] Two years and above

**10. What determines your choice of Hospital? *(Select all that apply)***

[ ] Proximity (location of health facility)

[ ] Working hours of health facility

[ ] Staff courtesy/friendliness

[ ] Specialized doctors/qualified health staff

[ ] Quality of ease of getting care/waiting time

[ ] Quality of facility cleanliness

[ ] Reputation of health facility

[ ] Cost of services

[ ] Others__________________

**11. In your opinion how serious should an ailment be before visiting a health facility?**

[ ] Life threatening

[ ] Very serious

[ ] Somewhat serious

[ ] Not serious

**12. What are the major diseases that has made you seek medical attention at a clinic or hospital in the past year**

[ ] Acute diseases (e.g. diarrhoeal, flu, malaria etc.).

[ ] Infectious diseases (TB, HIV/AIDS)

[ ] Chronic diseases (e.g. diabetes, hypertension, arthritis, etc.)

[ ] None of the above

[ ] All of the above

[ ] Others___________________

**SECTION -C: PREVENTIVE HEALTH CARE**

Please Tick [√] appropriately

**13. Are you aware of the term preventive health care?**

[ ] Yes

[ ] No

**14. If yes, where did you first learn about preventive healthcare? *(Tick all that apply)***

[ ] Mass media

[ ] Social media

[ ] Health workers

[ ] Family /friends

[ ] Religious/community leaders [ ] Others__________

**15. Which aspect of preventive healthcare do you prefer *(Tick all that apply)***

[ ] Interventions aimed at preventing a disease before they occur

[ ] Interventions aimed at reducing disease or injury impact

[ ] Interventions aimed at ameliorating the impact of on-going disease or injury with long lasting effect.

[ ] All of the above

[ ] None of the above

**16. Which of the following constitute preventive health care *(Tick all that apply)***

[ ] Preventive health check-ups/physical exams

[ ] Regular age related screening tests

[ ] Lifestyle- related advice like diet/nutritional counselling

[ ] Facilities for exercising like gym or park

[ ] Stress-relieving techniques like Yoga

[ ] Routine vaccinations

[ ] All of the above

[ ] None of the above

[ ] Others__________________

**17. How often or at what interval do you undergo preventive health check-up? *(Tick all that apply)***

[ ] Never

[ ] 6 monthly

[ ] Annually

[ ] Every two years

[ ] Every five years

**18. What preventive health measure do you take *(Tick all that apply)***

[ ] Immunization

[ ] Insecticide Treated Net

[ ] Exercise

[ ] Dieting

[ ] Stress management

[ ] Weight control

[ ] Yoga

[ ] Prophylaxis

**19. Which preventive healthcare services have you accessed in the past two years *(Tick all that apply)***

[ ] Mammography

[ ] Pap smear

[ ] BP check

[ ] Blood sugar

[ ] Cholesterol/lipids

[ ] Screening for STIs (including HIV)

[ ] Screening for Hepatitis B & C

[ ] Chlamydia screen

[ ] Colon cancer screen

[ ] Prostate cancer screen

[ ] Dental screen

[ ] Eye care

[ ] Weight check

[ ] Others (specify) --------------------

**20. Which of these would prevent you from accessing preventive health care? *(Tick all that apply)***

[ ] Poor access/distance to the healthcare providers

[ ] Cultural and religious beliefs

[ ] Underutilization of available health information

[ ] Inadequate/lack of Health insurance coverage

[ ] Stigma and social norms towards accessing certain health services

[ ] Inadequate education on benefits of Preventive Healthcare

[ ] Cost of healthcare

[ ] All of the above

[ ] None of the above

[ ] Others (please specify)……………….…....



**THANK YOU**

PARTICIPANT- All of my questions and concerns about this study has been addressed. I choose voluntarily to participate in this research project.

--------------------------------------------

Signature of Participant / Date

------------------------------------------------

Signature of investigator/ Date
